# Why Medical Students Choose to Use or Not to Use a Web-Based Electrocardiogram Learning Resource: Mixed Methods Study

**DOI:** 10.2196/12791

**Published:** 2019-07-11

**Authors:** Mikael Nilsson, Uno Fors, Jan Östergren, Gunilla Bolinder, Samuel Edelbring

**Affiliations:** 1 Section of Internal Medicine and Functional Area Emergency Medicine Department of Medicine Karolinska University Hospital Stockholm Sweden; 2 Department of Computer and Systems Sciences Stockholm University Stockholm Sweden; 3 Clinical Skills and Simulation Center Karolinska University Hospital Stockholm Sweden; 4 Faculty of Medicine and Health School of Health Sciences, Örebro University Örebro Sweden

**Keywords:** learning, medical, teaching, electrocardiogram

## Abstract

**Background:**

Electrocardiogram (ECG) interpretation is a core competence and can make a significant difference to patient outcomes. However, ECG interpretation is a complex skill to learn, and research has showed that students often lack enough competence. Web-based learning has been shown to be effective. However, little is known regarding why and how students use Web-based learning when offered in a blended learning situation.

**Objective:**

The aim of this paper was to study students’ use of Web-based ECG learning resources which has not previously been studied in relation to study strategies.

**Methods:**

A qualitative explanatory design using mixed methods was adopted to explore how medical students reason around their choice to use or not to use a Web-based ECG learning resource. Overall, 15 of 33 undergraduate medical students attending a course in clinical medicine were interviewed. Data on usage of the resource were obtained via the learning management system for all students. At the final examination, all the students answered a questionnaire on study strategies and questions about internet access and estimated their own skills in ECG interpretation. Furthermore, study strategies and use patterns were correlated with results from an ECG Objective Structured Clinical Examination (OSCE) and a written course examination.

**Results:**

In total, 2 themes were central in the students’ reasoning about usage of Web-based ECG: assessment of learning needs and planning according to learning goals. Reasons for using the Web resource were to train in skills, regarding it as a valuable complement to books and lectures. The main reasons for not using the resource were believing they already had good enough skills and a lack of awareness of its availability. Usage data showed that 21 students (63%) used the Web resource. Of these, 11 were *minimal users* and 10 were *major users* based on usage activity. Large variations were found in the time spent in different functional parts of the resource. No differences were found between users and nonusers regarding the OSCE score, final examination score, self-estimate of knowledge, or favoring self-regulated learning.

**Conclusions:**

To use or not to use a Web-based ECG learning resource is largely based on self-regulated learning aspects. Decisions to use such a resource are based on multifactorial aspects such as experiences during clinical rotations, former study experiences, and perceived learning needs. The students’ own judgment of whether there was a need for a Web-based resource to achieve the learning goals and to pass the examination was crucial for their decisions to use it or not. An increased understanding of students’ regulation of learning and awareness of variations in their ECG learning needs can contribute to the improvement of course design for blended learning of ECG contexts for medical students.

## Introduction

### Background

Web-based learning has been shown to be effective when implemented as a primary mode of teaching or as a complementary resource in a blended learning setting [1,2]. The advantages of Web-based complementary resources are user control over content, learning sequence, pace, and chosen time to study. However, other strengths of a computer resource have been described in the study by Bond et al [3]. This flexibility allows learners to adapt their usage to meet course objectives and personal learning objectives in varying medical education contexts [4].

Knowledge in electrocardiogram (ECG) interpretation is a core competence for physicians and can make a significant difference to patient outcomes [5,6]. However, ECG interpretation is a complex skill to learn, and research has shown that some students have a lack of competence [7]. There is an ongoing discussion about how to perform effective ECG teaching. A recent review showed that no single method or format of teaching is superior in enhancing ECG interpretation skills [8], thereby implying a need for flexible ECG learning activities. Most studies regarding ECG education include reports of skill shortages, measurements of education or student experience, or demonstration of opportunities with a type of education. Thus, they do not help to understand why and to what extent students make use of various educational resources. In the evaluation of Web-based resources, the importance of the student perspective has been stressed [9]. According to Illeris, learning can be studied from different perspectives: environment, content, and incentive [10]. From the incentive perspective, it is important to understand how students perceive the content and what drives their choice of ECG learning resources. Students’ use of Web-based ECG learning resources has not previously been studied in relation to study strategies. To our knowledge, students’ decision-making process has not been studied in settings where students have the opportunity to use a Web-based complementary ECG learning resource. Theories of self-regulated learning shed light on students’ incentives and approaches to flexible learning tools [11,12]. The self-regulated learning process is described as being directed by monitoring one’s own learning needs and using necessary tools to support the learning process [13-15]. Brydges and Butler argue that self-regulation should be studied and understood across a variety of learning contexts available in medical education [16]. Using the self-regulated learning perspective to understand the use of a Web-based ECG learning resource means taking the learners’ perspective as the point of departure.

### Study Aim

The primary aim of this study was to explore medical students’ rationales for choosing to use a Web-based supplementary resource for ECG learning. With this aim, we conducted a mixed methods study.

## Methods

### Overview

**Figure 1:**
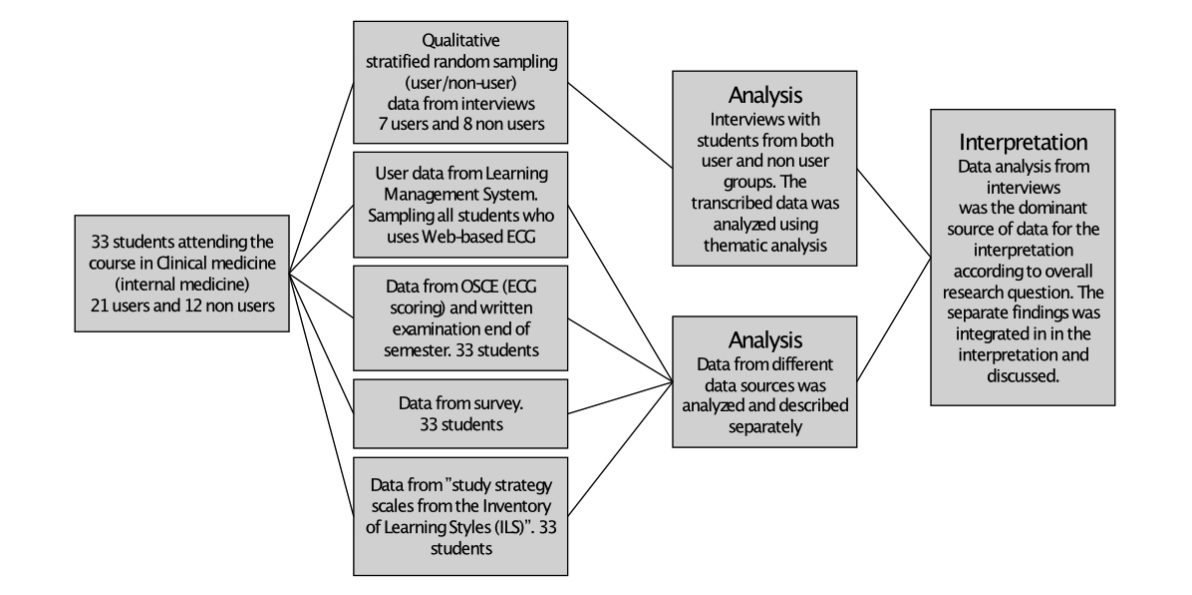
Flowchart of the study design. In total, 33 students attended the course in internal medicine and received information about the study and introduction to the Web-based electrocardiogram (ECG) learning resource. Of the 33 students, 21 chose to use the resource. For those students, the learning management system collected usage data. Students were chosen alphabetically to be interviewed. After 15 interviews, data saturation was achieved. In addition to the course examination, students performed an ECG interpretation Objective Structured Clinical Examination (OSCE) station. Results from the OSCE station and final examination were collected. During the examination, students were asked to fill in a questionnaire, which all 33 students completed.

In this exploratory mixed methods study (see Figure 1), we used a combination of interviews and quantitative data. Data analysis from interviews is the dominant source of data for interpretation according to the overall research question. By using a qualitative method, we can, through interviews, explore in a deeper way the decision-making process and the reasons for using Web-based education. At the same time, we wanted to investigate whether there are any objective data that reinforce the possible model that students use in decision making. This can be achieved by comparing results from questionnaires and from examinations at the group level.

The study group comprised all third-year medical students studying internal medicine at 1 of 4 teaching hospitals at Karolinska Institutet (KI), Stockholm, Sweden. All students had passed a basic course in ECG interpretation 2 semesters before the period under study. The course in internal medicine combines theory and practice, in that approximately 50% of the course consists of lectures and seminars, and the other part is spent in clinical rotations.

All students were given access to a Web-based ECG learning program 8 weeks before their final examination. Development and structure of the Web-based ECG resource has been described in detail elsewhere [17,18].

A part of the course examination was an Objective Structured Clinical Examination (OSCE) test with 1 station examining ECG interpretation skills. The students received an email explaining the study and how they could access the Web-based ECG learning program approximately 8 weeks before the OSCE. The email contained detailed information on program structure but no directions about how to plan learning activities. The examiner (coauthor JÖ) also discussed the program during a lecture. The regional ethics review board in Stockholm approved the study (2009245-314).

A total of 33 students attending the course in internal medicine received information about the study and introduction to the Web-based ECG learning resource. Of the 33 students, 21 chose to use the resource. For those students, the learning management system collected usage data. Students were chosen alphabetically to be interviewed. After 15 interviews, data saturation was achieved. In addition to the course examination, students performed an ECG interpretation OSCE station. Results from the OSCE station and final examination were collected. During the examination, students were asked to fill in a questionnaire, which all 33 students completed.

### Interviews

An interview guide was created based on pilot interviews with 7 students. From the group of the 33 medical students, 15 participants were selected to be interviewed (Figure 1). The intention was to interview both students who used and did not use the Web-based program. Students were selected alphabetically from the course list, adjusting for equal numbers of users and nonusers of the Web-based program. Overall, 7 users (4 women and 5 men) and 8 nonusers (2 women and 6 men) were interviewed by the first author or a student administrator 3 months after the examination. The first interview was performed collaboratively by the author and the administrator to synchronize the interviewers and make final adjustments to the interview guide. Students were informed that both interviewers were independent from the course management and that participation or nonparticipation in the study would not affect their grades. During the interview, students were asked to share their thoughts and reasoning behind their choices of using or not using the Web-based program. They were also asked to share general thoughts about Web-based learning and traditional media, such as textbooks and lecture notes. Furthermore, the students were asked to explain if and how they used the Web-based ECG learning program. The semistructured interviews were completed by telephone, recorded digitally, and transcribed verbatim.

### Analysis of the Interviews

Data were analyzed using thematic analysis ([16,18]. In addition, 2 of the authors (MN and UF) performed the primary analyses, which were then discussed with the other authors. Initial readings of the transcribed texts were then coded and grouped according to the research question. The codes were analyzed for variability, consistency, and emerging patterns. The final codes were analyzed iteratively in a process of reading and rereading, leading to broader themes. The themes are exemplified by transcript quotations.

### Questionnaire

All 33 students answered a questionnaire on the final examination day. The first part consisted of an estimation of ECG interpretation knowledge in relation to the course objectives and the availability of computer and internet access; the second part consisted of questions about individual study strategies. For the latter part, study strategy scales from the Inventory of Learning Styles (ILS) by Vermunt were used [19]. The regulation strategy scales consist of 28 items forming the 3 variables: *self-regulated*, *external regulated,* or *lack of learning strategies*.

The self-regulated items relate to how students plan learning activities, how they test their progress, and how they direct themselves toward self-generated learning objectives. The external regulated items relate to how students may let themselves be led by didactic aids, such as learning objectives, assignments, or teacher-/supervisor-generated questions. The lack of learning strategy items relates to how students may have problems assessing mastery and comprehension or lack clear ideas about relevant objectives and problem-solving approaches.

The scales have previously been successfully used in medical studies as well as in other higher-education student groups [14,20]. The Swedish translation of the scales has previously been validated in a Swedish medical context [20].

We also collected user activity logs from the learning management system used by KI (Ping Pong) during the time the students used the Web-based ECG learning program.

An *active user* was defined as a student who had been logged on for at least 30 min in the system. For further analyses, the user group was divided into 2 parts based on the median user time of 2 hours and 46 min. Group 1 students logged in less than the median time, and group 2 students logged in for the median time or longer.

The OSCE included 2 ECGs representing life-threatening conditions. A total of 20 points were distributed for correct interpretation, with 12 points for an ECG showing an ST-elevation myocardial infarction with atrial fibrillation and 8 points for an ECG showing ventricular tachycardia. The final written examination contained questions covering the entire field of internal medicine with a maximum of 100 points given.

### Statistical Analysis

We used SPSS 17.0 for descriptive data calculations. Time was described in hours and minutes (h:mm). Tests of normality for OSCE ECG were done using both Schapiro-Wilk and Skewness methods showing non-normal distribution data.

The level of statistical significance was set to *P*<.05. All statistical tests were 2-sided. Correlations were measured by the Spearman rho. Cronbach alpha was calculated for each scale of the ILS using the SAS System 9.1.

## Results

### Interviews

**Figure 2:**
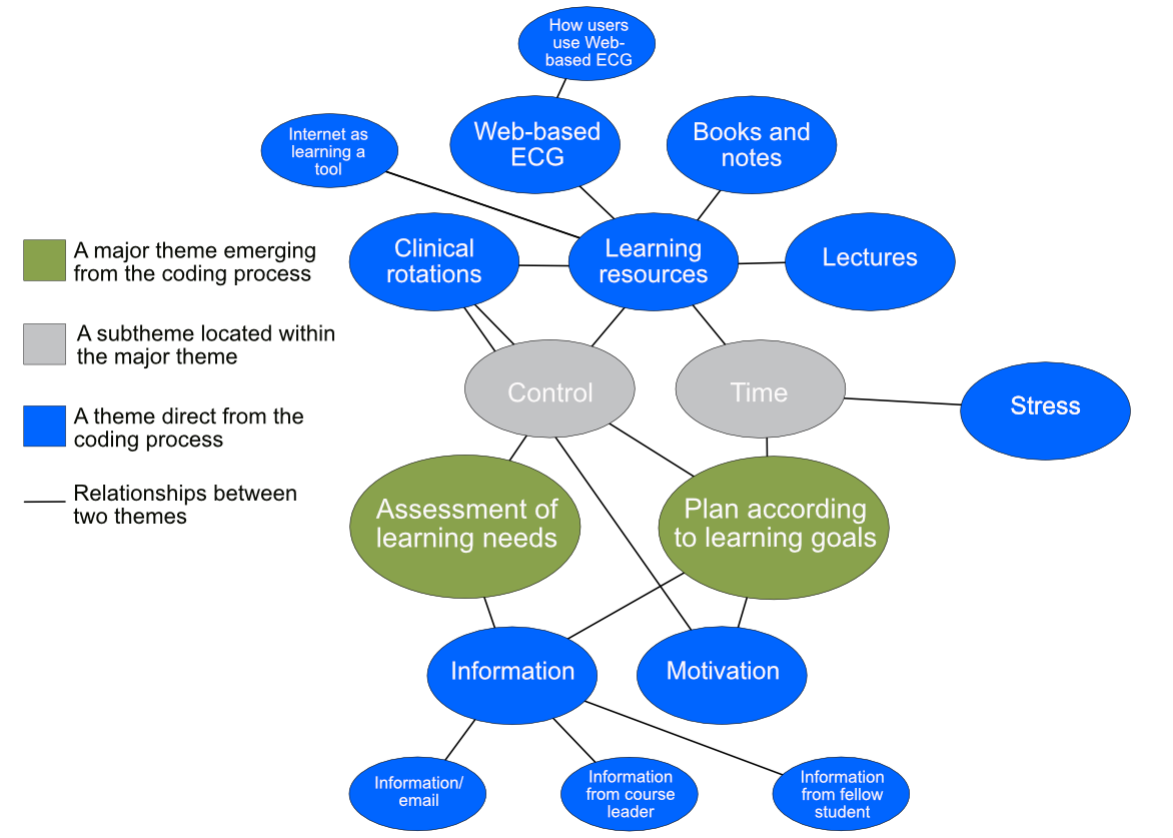
Resulting themes and their relationships after interview analysis. Blue objects represent themes derived directly from interviews and connect either to another “blue” theme or directly to an overall perspective. Two important themes—assessment of learning needs and plan according to learning goals—are key factors affecting the decision to use or not use the Web-based learning resource. ECG: electrocardiogram.

In the thematic analysis of the 15 interviewed students, 2 overarching themes were identified: *assessment of learning needs* and *planning according to learning goals*. Figure 2 describes a thematic map of the interview results.

#### Assessment of Learning Needs

All students considered ECG interpretation to be important in their future roles as physicians. All students also identified ECG interpretation as a learning objective of the ongoing course in internal medicine as well as part of the OSCE and written examinations. Assessment of learning needs was a consistent theme for the majority of students. The students talked about it sometimes as more of an intuitive feeling but more often as a conscious process involving concrete interaction with some practical experience of control from other persons or self-control. Most students described assessment of learning needs as a recurrent theme involving the other central theme, *planning according to learning goals*. Assessment of learning needs is associated with 2 subthemes: *information* and *control*.

##### Information to Students About the Web-Based Program

Students reported receiving information from email and verbal information from the examiner (JÖ) about the availability of the Web resource. Several students believed this was important information from the examiner and a reminder of the previous information via email. Email information was perceived to be useful for some students. Occasionally, students learned about the Web-based program through friends. One student reported finding the program via the learning management system and forgot he had received the information by email:

My course director (JÖ) strongly encouraged us to use the system. He was responsible for the course. He’s an authority that you listen to, but he also came especially to a lecture to inform.

S16

First, we got the information through email, but then JÖ also informed after a lecture, so we got the information in two different ways...

S3

I forgot most of it anyway when we receive information by email, it’s so much mail. Maybe not daily, but say, it will be two school-related emails a day then it will be like you are drowning in information there...

S1

##### Control and Assessment of Learning Needs

The students related to control in interaction with the main themes and sometimes as a loop with learning resources. Practical experiences included assessing ECGs during clinical rotations and using the Web-based program. The Web-based program was thought to stimulate control for users by making ECG cases and formative tests available.

Clinical rotations were thought to be important for the students, both in evaluating their knowledge of ECG interpretation in clinical practice and as an important learning resource. Students reported experiences during clinical rotations as an important part of their decision about how to learn to interpret ECGs. Many reported a perceived lack of *good enough* knowledge in ECG interpretation in the clinical environment as a major reason for using the Web-based program. For most students, usage was not affected by access to books on ECG interpretation, lecture notes, and exercise examples of ECG.

##### Users

Both users and nonusers affirmed the importance of repetition to reach the learning objectives. The user group described a need for repetition and thus chose to use the Web program:

Because I felt that I had forgotten too much of the previous ECG teaching. I needed to brush up skills... We had a lot of experience from clinical rotations, when you stand there and look at an ECG and were asked: What do you see? How do you interpret this? So I felt that I had lack of knowledge… The test at the basic ECG-course in the semester before went great, but because we don’t use the knowledge so much in a period I lost quite a lot, and then I felt that I needed that refresher...

S17

##### Nonusers

Some students had a positive learning experience during the rotation in cardiology and, afterward, felt only a minor need for repetition with their books or other study material. Although most students had a positive attitude toward internet resources for studies and social contacts, some students did not like using the internet for learning or for social contacts:

Right then I did not see the need (for the Web-based program). I used the book I got for the clinical diagnostics course of ECG interpretation and a book with examples. It was sufficient for me. So quite honestly, I was not even inside and watched the Web-based training but I knew it was there...

S12

I looked at old exams and things like that so I know that everything was OK. I will probably get through the course with a passing grade.

S12

#### Planning According to Learning Goals

Planning according to learning goals was a consistent theme. The students talked about it as a conscious process in interaction with the other central theme, *assessment of learning needs*. Planning according to learning goals is associated with 2 themes: *time* and *control*. The other associations also mentioned were information and motivation.

##### Time

The curriculum is extensive, and the students described a need to plan their studies. Some students mentioned that their knowledge in ECG interpretation had been very good from a previous course in clinical diagnostics, but their knowledge had declined markedly.

Students described various clinical rotations during which they were offered a Web-based program. The time lag after the course in clinical diagnostics was reported as a reason for requiring repetition. A few students had a prolonged time lag because of a pause in studies for reasons such as maternity leave, further amplifying the need for repetition.

##### Nonusers

Nonusers often described lack of time or sometimes a more negative attitude to computer-based training as contributors to their decisions not to use the program:

No, I have not used it. No, I do not really like this computer-based training and it doesn’t work so well for me...

S2

No, I learn more from reading a real book that you can hold on and sit back and flip in...

S2

Then I didn’t practice so much on the ECG, it was more a matter to look up single items, but those I knew already where they were in the book...

S2

##### Users

Users mentioned the need for repetition as an important element, and they saw the usefulness of the program to reach the learning goal:

Because I had a need of repetition and it seemed like an easy way to repeat it...

S3

Because I was nervous before the exam, probably. Had I missed something, or forgot something. A good repetition...

S4

#### How Users Use the Program

Students reported using the program to varying extents and in different ways. Most students used the interactive parts of the ECG cases. Some students described that they used the program sequentially, in an A-to-Z manner. Other students jumped back and forth between different parts of the program. Some students felt they needed more time to repeat or learn ECG interpretation than was available:

I completed some of the self-assessment questions, I then mostly practiced with interactive interpretations...

S16

I probably followed all the steps pretty accurately. I read the information contained in each section. Then I did the test that was linked to as well to see that I had understood...

S17

I guess I thought everything in the program was good. I cannot remember that I felt annoyed that anything was strange or wrong, I thought it was great that one could, only assimilate information and then directly get confirmation that one had known what it was about...

S17

I used mainly the ECG interpretation training section. The other parts I did not check very much, I thought I had read it enough recently and did not have enough time to sit down and go through them more carefully.

S15

### Questionnaire

All 33 students participated in the survey and completed the OSCE and final general examination. The participants included 16 women (48%) and 17 men (52%). All students had access to a broadband internet connection, making it possible to run the Web system if they wanted.

A total of 21 (64%) students were classified as users (>30 min of usage) of the Web-based resource. Median time that users logged onto the system was 2:46 (h:mm, interquartile range [IQR] 1:28-6:37). In addition, 57% of the users were women compared with 33% women in the nonuser groups. The user and nonuser groups were similar regarding their results at the ECG question at the OSCE station and the final examination (Table 1). Self-estimated knowledge of ECG interpretation and learning strategy was also similar between groups.

We further divided the users into 2 groups based on the 2:46 median time value of the user group (Table 2). Among them, 12 students were determined as minor users (median time 1:34, IQR 0:47-2:17) and 9 students were major users (median time 6:38, IQR 5:12-9:21). The major user group included 8 females and 1 male. There was a difference in performance on the ECG test in the OSCE (median females 18.0 p, IQR 16.0 p-18.8 p; median males 16 p, IQR 14.5 p-16.5 p; *P*<.001), but a gender difference was not seen in the final general examination (females median 74.5, IQR 69.2-80.9; males median 74.0, IQR 68.8-77.5; *P*=.68).

**Table 1:** Student characteristics, self-ratings, scores in the electrocardiogram (ECG) Objective Structured Clinical Examination (OSCE), final general examination scores, and results from strategy scales from the Inventory of Learning Styles.

Characteristics		User	Nonuser
Total students, n (%)		21 (64)	12 (36)
**Gender, n (%)**			
	Female	12 (75)	4 (25)
	Male	9 (53)	8 (47)
Total activity in Web-based ECG learning resource time (h:mm), median		2:46	—^a^
OSCE ECG test, median points		16	16
Final general examination, median points		74.5	71.8
Students estimated their knowledge of ECG interpretation as 0%-100% of the course objectives, median percentage		80	80
Self-regulation scale, median		3	3
External regulation, median		3	3
Lack of regulation, median		2	2

^a^Not applicable.

**Table 2:** Student characteristics, self-ratings, activity in Web-based electrocardiogram (ECG) learning resource, and results from strategy scales from the Inventory of Learning Styles.

Characteristics		Minor user (30 min-2:46 h)	Major user (>2:46 h)
Total students, n (%)		12 (57)	9 (43)
**Gender, n (%)**			
	Female	4 (33)	8 (67)
	Male	8 (89)	1 (11)
Total activity in Web-based ECG learning resource time (h:mm), median		1:38	6:38
OSCE^a^ ECG test, median points		16	18
Final general examination, median points		74.5	74.5
Students estimate their knowledge of ECG interpretation 0-100% of course objectives, median percentage		80	85
Self-regulation, median		3	3
External regulation, median		3	3
Lack of regulation, median		2	2

^a^OSCE: Objective Structured Clinical Examination.

Table 3 shows results from the Spearman rank-order correlation between ECG results in OSCE, activity in the Web-based ECG learning resource, and strategy scales from the ILS. There was no correlation between the OSCE results and activity time in the Web-based ECG resource (Table 3). We also tested for association between the regulation strategy scales (self-regulated, externally regulated, or lack of learning strategies). There was a correlation between OSCE results and self-regulation (r_s_=0.37; *P*=.03), as well as a negative correlation between OSCE results and lack of regulation (r_s_=−0.56; *P*=.004). No correlation was seen between OSCE results and external regulation. There was also no correlation between regulation strategy scales and time in interactive ECG interpretation (Table 3).

**Figure 3:**
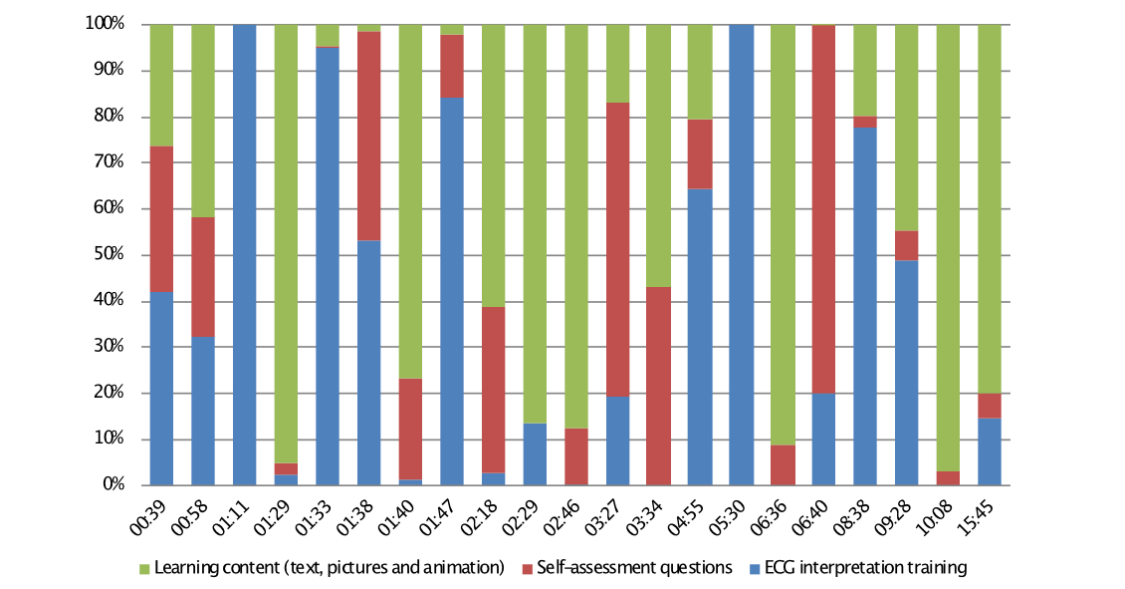
The 21 students’ distribution of time in each activity in the Web-based ECG learning resource. Each bar represents 1 student. Total time=hh:mm. ECG: electrocardiogram.

Figure 3 shows the distribution of time in each part of the Web-based ECG program for all users based on server logs.

**Table 3:** Results from the Spearman rank-order correlation between electrocardiogram result in Objective Structured Clinical Examination, activity in Web-based electrocardiogram learning resource, and strategy scales from the Inventory of Learning Styles.

Variable	Spearman rho	*P* value
OSCE^a^ result and total activity in Web-based ECG^b^ learning resource	.14	.52
OSCE result and time in interactive ECG interpretation	.29	.20
OSCE result and self-regulation	.37	.03^c^
OSCE result and external regulation	.10	.58
OSCE result and lack of regulation	−.56	<.001^c^
Time in interactive ECG interpretation and self-regulation	−.11	.64
Time in interactive ECG interpretation and external regulation	−.06	.80
Time in interactive ECG interpretation and Lack of regulation	−.20	.36

^a^OSCE: Objective Structured Clinical Examination.

^b^ECG: electrocardiogram.

^c^Statistical significance *P*<.05.

## Discussion

### Overview

**Figure 4:**
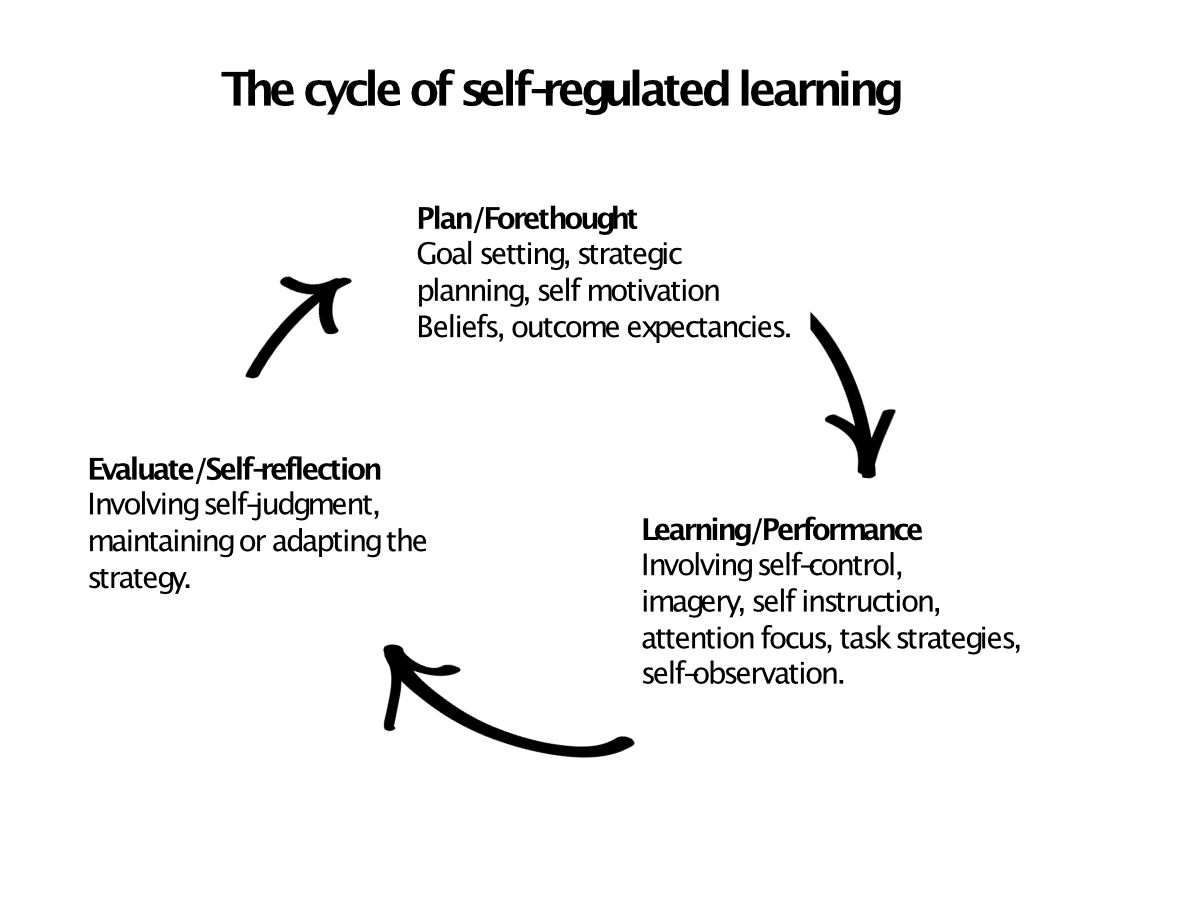
The cycle of self-regulated learning adapted from Zimmerman [21]. The student's were active in constructing their own meanings and goals from various influences (clinical rotations and earlier experience of electrocardiogram [ECG] examinations). The individuals were capable of monitoring and controlling various aspects of learning (from fellow students, teachers, and the Web-based ECG). Individuals set goals for their learning, and monitored the learning process towards these goals (clinical rotations, testing knowledge from old course examinations).

In this study, we explored how medical students decided to use or not to use a Web-based ECG learning resource in a blended learning situation. The findings suggest a pattern largely driven by students’ incentives in a self-regulated learning process (Figure 4; adapted from Zimmerman [21]).

The interviews highlighted different aspects behind the students’ decisions to what extent, and how, they used (or did not use) the Web-based training resource. To plan their own learning or decide what resources they should use for acquiring or refreshing skills in ECG interpretation, the students seemed to use 2 overarching questions to regulate their use of available resources. First, what is my current level of knowledge? Second, what learning outcomes must I reach?

Students seemed to have a sense of their own level of ECG interpretation skills based on their past experiences from clinical rotations, old lecture notes, and more recent lectures during the course. The clinical rotations seemed to be an important opportunity for students to discover their actual levels of knowledge. On the basis of this, the students continued using the accessible learning resources to reach their learning goals. A majority of all the students were positive toward Web-based education in general as an on-demand resource. The users were positive to the Web-based ECG resource, which is in line with our previous results [17,18]. A quality measure and one of the factors that contributed to the decision to use the Web-based program was influence from fellow students who used the resource or if a teacher spoke positively about the program. According to Illeris’ learning model, all learning will always involve the 3 different perspectives: environment, content, and incentive [10], which is in line with our findings. However, the interviews show an emphasis on the *incentive* dimension in Illeris’ learning model. The above-described interaction between the student, a fellow student, and/or the teacher in the decision-making process represents the *incentive* dimension interacting with the *environment* dimension. Being able to integrate various social influences from the environment may be an important ability in the process of self-regulated learning [16].

The students were active in constructing their own meanings and goals from various influences (clinical rotations and earlier experience of ECG examinations). The individuals were capable of monitoring and controlling various aspects of learning (from fellow students, teachers, and Web-based ECG). Individuals set goals for their learning and monitored the learning process toward these goals (clinical rotations and testing knowledge from old course examinations).

Winters et al emphasized the control of learning as an important factor for students in the context of computer-based learning [11]. The students presented evidence that different learner and task characteristics (eg, previous knowledge, goal orientation, and learner control) and types of learner support are related to self-regulated learning when using computer-based learning. Our results are in line with previous studies showing that higher abilities of self-regulated learning are linked to better academic performance [22,23], as students with higher scores on *self-regulation* had better results at the OSCE station, whereas students scoring high on *lack of regulation* had lower scores at the OSCE (Table 3).

However, the level of performance (high vs low) in these strategies did not influence the use of the Web-based ECG learning resource. There was no difference in OSCE results between groups of users and nonusers of the Web-based resource, which is in line with the pattern that emerged in the interviews, suggesting a process of self-regulated learning based on learning needs instead of preference for a certain learning strategy. Interview data showed that students do not seem to prioritize *overlearning* as a learning objective before the written general examination and the OSCE. Their goal in general was not to maximize knowledge but to pass the examinations. In Swedish medical education, there is only a pass/fail grade, which could possibly explain this pattern.

Our observed lack of correlation between the time using the system and the results at the OSCE test strengthens the findings from the interviews that students plan their own learning and decide what resources they should use for acquiring or refreshing skills in ECG interpretation. These findings also partly confirm data from a similar context in a blended learning situation in medical education. In a study of online volume training of interpreting ECG strips, there was no clear relation between the number of ECGs studied during the training period and marks obtained by medical students in the examination [24].

In a recent review, no single method or teaching format was considered more effective than the others in delivering ECG interpretation knowledge [8]. However, research considering self-studies in learning ECG interpretation shows contradictory results. In a controlled study, the authors found lower test results in the self-study group compared with other forms of study methods [25]. In contrast, Kopec et al found that ECG knowledge in students during the last year of medical education was superior in the student group who used self-studies to learn ECG. Our data suggest that in a realistic training situation, it is not the primary method of learning that is decisive but the ability to use available methods based on motivation. From this perspective, the findings from the study by Raupach et al become interesting. They found that the students’ valuation of an assessment affected the gained knowledge more than the specific method itself [26].

### How the Students Used the Web-Based Electrocardiogram Learning Program

The differences in usage patterns of the Web resource were large, as illustrated by our log-file analysis. Users occasionally changed patterns of use after some time spent in the system. The main reason, according to the interviews, is lack of time. In a previous study, the choice to use the Web-based ECG learning program was not related to individual learning styles [18].

Further research is needed to identify why students, from a self-regulating perspective, chose to use different parts of the Web-based learning content (text, pictures, and animation), self-assessment questions, and an interactive ECG interpretation training section. A better insight into students’ general learning strategies and increased awareness of learners’ specific needs of ECG skills can improve further design of blended ECG learning contexts.

### Limitations

There are 2 major limitations to this study. First, the OSCE test contained only 2 ECGs, so the ability to differentiate ECG knowledge was limited. Second, the students’ activity in each part of the ECG program was measured through a learning management system. Although the system logged out when idle, we cannot know with certainty how active students were during training sessions in the program.

### Strengths of the Study

In this study, we used a mix of qualitative and quantitative methods to achieve a broader perspective and a nuanced picture. The interviews contributed depth and provided rationales for the students’ choices and their strategies on how to use, or not use, the Web-based ECG program.

### Conclusions

A supplementary Web-based ECG resource contributes to student learning based on principles of self-regulated learning in which students make their decisions based on a multitude of factors. These factors include experiences during clinical rotations, former study experiences, and their individual strategy for regulating their learning. An overarching aspect of usage of the resource is the relation to individual learning goals and needs to pass the examination was the students’ judgment of whether there was a need for a Web-based resource to achieve their learning goals. On the basis of individual variations, the usage patterns of ECG resources are not predictable. However, a better understanding of variations in regulating learning and perceived needs of ECG knowledge can improve the course design of blended learning ECG contexts for medical students.

## Abbreviations

ECG: electrocardiogram
ILS: Inventory of Learning Styles
IQR: interquartile range
KI: Karolinska Institutet
OSCE: Objective Structured Clinical Examination
